# Validation of the Persian version of the dysphagia handicap index in patients with neurological disorders

**Published:** 2016-07-06

**Authors:** Ebrahim Barzegar-Bafrooei, Jalal Bakhtiary, Ahmad Reza Khatoonabadi, Farzad Fatehi, Saman Maroufizadeh, Mojtaba Fathali

**Affiliations:** 1 Department of Speech Therapy, School of Rehabilitation, Tehran University of Medical Sciences, Tehran, Iran; 2 Department of Speech Therapy, School of Rehabilitation, Semnan University of Medical Sciences, Semnan, Iran; 3 Iranian Center of Neurological Research, Department of Neurology, Shariati Hospital, Tehran University of Medical Sciences, Tehran, Iran; 4 Department of Epidemiology and Reproductive Health, Reproductive Epidemiology Research Center, Royan Institute for Reproductive Biomedicine, Academic Center for Education, Culture and Research, Tehran, Iran; 5 Department of Ear, Nose and Throat, School of Rehabilitation, Tehran University of Medical Sciences, Tehran, Iran

**Keywords:** Dysphagia, Disability Evaluations, Persian, Iran, Reliability and Validity, Neurologic Disorders, Reproducibility of Results, Questionnaire Design

## Abstract

**Background:** Dysphagia as a common condition affecting many aspects of the patient’s life. The Dysphagia Handicap Index (DHI) is a reliable self-reported questionnaire developed specifically to measure the impact of dysphagia on the patient’s quality of life. The aim of this study was to translate the questionnaire to Persian and to measure its validity and reliability in patients with neurogenic oropharyngeal dysphagia.

**Methods:** A formal forward-backward translation of DHI was performed based on the guidelines for the cross-cultural adaptation of self-report measures. A total of 57 patients with neurogenic dysphagia who were referred to the neurology clinics of Tehran University of Medical Sciences, Iran, participated in this study. Internal consistency reliability of the DHI was examined using Cronbach’s alpha, and test-retest reliability of the scale was evaluated using intraclass correlation coefficient (ICC).

**Results:** The internal consistency of the Persian DHI (P-DHI) was considered to be good; Cronbach’s alpha coefficient for the total P-DHI was 0.88. The test-retest reliability for the total and three subscales of the P-DHI ranged from 0.95 to 0.98 using ICC.

**Conclusion:** The P-DHI demonstrated a good reliability, and it can be a valid instrument for evaluating the dysphagia effects on quality of life among Persian language population.

## Introduction

Oropharyngeal dysphagia is defined as a difficulty in moving bolus from the mouth to the stomach due to neurologic, structural, or other medical conditions. It commonly occurs in 30-84% of individuals with neurological disorders [i.e., stroke, Parkinson’s, amyotrophic lateral sclerosis (ALS), and multiple sclerosis (MS)].^1^ The abnormal swallowing can lead to malnutrition and weight loss, dehydration, aspiration pneumonia, and even death.^2^^-^^5^ Furthermore, the inability to eat represents a social handicap that not only affects the patient physically but also mentally.^6^ Dysphagia can interfere with many aspects of one’s life including work, leisure, social interactions, and self-esteem. It ultimately leads to reduced quality of life for the patient and those around him or her.^6^^-^^9^

In recent years, several questionnaires were designed to assess quality of life in the patients with dysphagia, but most of them are disease-specific. In 2001, Chen et al.^10^ created the M. D. Anderson Dysphagia Inventory to evaluate quality of life-related to dysphagia in the patients with head and neck cancer. Woisard et al.,^11^ in 2005, developed the Deglutition Handicap Index, which is a patient-reported 30-item questionnaire that measures the deglutition-related aspects in daily life and is a valid tool. The dysphagia-goal-handicap (DGH) was developed to examine the handicapping effect of esophageal dysphagia in 1991. It was indicated that dysphagia affects quality of life.^12^ One of the comprehensive patient-reported tools for dysphagia is the swallowing quality of life questionnaire which has more popularity in research areas of this regard. However, the completion of this test is time-consuming and understanding some of its items is difficult for the patient. Thus, its application is limited to clinical practice.^13^

In 2012, Dysphagia Handicap Index (DHI) was developed by Silbergleit et al.^7^ The purpose of creating DHI was to provide a tool that can measure dysphagia disabling effects on the physical, functional and emotional aspects of individual’s life in a wide variety of swallowing disorders.

DHI is a new and efficient tool that has excellent psychometric characteristics and can be used in planning or modifying treatment approaches.^7^ It has been translated into Arabic. An Arabic translation and validation of this questionnaire have shown good psychometric properties;^14^ however, this test has not been translated to Persian. Regarding the high prevalence of oropharyngeal dysphagia following stroke and other neurological disorders,^1^ the purpose of this study was to translate DHI into Persian and to evaluate its validity, internal consistency and test-retest reliability in neurogenic oropharyngeal dysphagia.

## Materials and Methods

***Persian-DHI (P-DHI) translation process***

The process of developing P-DHI was performed based on the guidelines for the cross-cultural adaptation of self-report measures in five steps.^15^ First, the original version of DHI was translated separately by two translators into Persian. Second, a meeting was held with speech pathologists and translators to investigate Persian translations of DHI and prepare preliminary Persian version of the index. In the third stage, the preliminary Persian version of the index was back-translated into English by two other translators and was approved by the original author. In the fourth stage, a meeting was held to prepare pre-final Persian version of DHI (P-DHI). In the fifth step, the pre-final Persian version of the index was completed tentatively by patients with dysphagia, and finally after considering the views of experts and participants, the final P-DHI was developed. All of the items on the DHI were directly translated into Persian and received no changes.

***Participants***

This study was performed in teaching hospitals of Tehran University of Medical Sciences, Iran, in 2014. 57 consecutive adult Persian patients with oropharyngeal dysphagia were included in this study. Patients participated in this study based on the diagnosis of their specific neurological disorders including stroke, Parkinson’s disease, ALS, brain tumors, MS, and myasthenia gravis. All patients were evaluated by the Northwestern Dysphagia Patient Check Sheet^16^ and provided that the results of this test showed that the patient had at least one of the disorders of deglutition phase (oral phase or pharyngeal phase) or, aspiration, or pharyngeal delay could enter this study. Exclusion criteria were (1) inability to understand written or spoken Persian, (2) evidence of purely esophageal dysphagia, (3) evidence of cognitive problems as screened by the Mini-Mental State Examination (MMSE) (a score of < 23).^17^ And finally, to measure the reliability of test-retest, the P-DHI was completed by 14 patients twice in a period ranging from 1 to 2 weeks. During this period, the patients received no medical, surgical or behavioral intervention for swallowing disorders.

***DHI questionnaire*** ^7^

The DHI is composed of 25 items and three physical, functional, and emotional subscales. 

**Table 1:** Features of Dysphagia Handicap Index (DHI) subscale distributions of the patient group

**DHI scale**	**Number of items**	**Possible range**	**Observed range**	**Mean ± SD**
Physical	9	0-36	32-2	13.28 ± 6.90
Functional	9	0-36	32-0	11.47 ± 7.61
Emotional	7	0-28	0-26	9.75 ± 5.11
Total	25	0-100	10-78	34.51 ± 16.35

DHI: Dysphagia Handicap Index; SD: Standard deviation

The physical subscale includes 9 items that represent the individual’s perception of physical discomfort caused by dysphagia. The emotional subscale is composed of 7 items that examine patient’s emotional reactions to his dysphagia and functional subscale includes 9 items which are related to the impact of dysphagia on daily activities of a person’s life. For each question, three answers are considered (never, sometimes and always) that are scored (0, 2 and 4, respectively). After completing the test by the patient, subjects are asked to measure their severity of dysphagia by a 7-point equal-appearing interval scale. On this scale, number 1 represents no problem as number 7 represents a serious one and number 4 shows moderate dysphagia.

***Validation and statistical testing***

The P-DHI was validated using content validity. The P-DHI and the back translated version presented to five swallowing therapist. They scored each question based on the quality of translation, fluency, understandability, and the cultural context.

Internal consistency of the P-DHI was examined using Cronbach’s alpha, and test-retest reliability of the scale was evaluated using intraclass correlation coefficient (ICC). To compare mean P-DHI subscales and total scores with the self-reported dysphagia severity, one-way ANOVA and Duncan post-hoc test were used. Spearman’s correlation coefficient was also used to evaluate the relationship between the total P-DHI scores, P-DHI subscale scores, and the self-reported dysphagia severity scores. Data were analyzed using SPSS software (version 17, SPSS Inc., Chicago, IL, USA). All statistical tests were two-tailed and a P < 0.050 was considered statistically significant. The data are presented as a mean ± standard deviation (SD).

## Results

A total of 57 patients with neurological disorders, 21 males and 36 females participated in this study (mean age 54.96 ± 14.81 years). Table 1 shows a range of the total P-DHI and its subscales scores in patients under the study (Table 1). The mean total P-DHI score of the patients was 34.51 ± 16.35. 

The content validity of the P-DHI was also evaluated; the fluency, understandability and cultural adaptation of the P-DHI confirmed by swallowing therapists and patients.

To assess the internal consistency of the scale, Cronbach’s alpha coefficient was calculated for each subscale and also the total (Table 2). Cronbach’s alpha coefficient was (α = 0.88) for the total scale and demonstrated good internal consistency of the scale items The ICC for the total P-DHI and physical, functional and emotional subscales were between 0.95 and 0.98 (Table 2). 

**Table 2:** Internal consistency and test-retest reliability of the Persian Dysphagia Handicap Index (P-DHI)

**DHI scale**	**Cronbach’s alpha ** **(n = 57)**	**ICC ** **(n = 14)**
Physical	0.73	0.95
Functional	0.83	0.97
Emotional	0.71	0.96
Total	0.88	0.98

DHI: Dysphagia Handicap Index; ICC: Intraclass correlation coefficient

Adapted from the original version of DHI, the self-reported severity of dysphagia scale was grouped into four categories: 1: normal; 2 and 3: mild; 4 and 5: moderate; 6 and 7: severe. The mean scores of total P-DHI and its subscales for the severity groups are presented in table 3. Data analysis showed that there is a significant difference between the four severity groups considering the P-DHI and the three subscales (P > 0.001).

The results of post-hoc analyses of the severity groups showed that all the pairwise comparisons were significant, except for the normal and mild severity groups (P > 0.050). Spearman’s correlation coefficient was calculated to assess the relationship between P-DHI scores and the self-reported dysphagia severity scores. The results indicated that there is a significant relationship between the total score (r = 0.67, P < 0.001) and the subscales (physical, r = 0.59, P < 0.001; functional, r = 0.49, P < 0.001; and emotional, r = 0.52, P < 0.001).

**Table 3:** Dysphagia Handicap Index (DHI) subscales and total DHI for the self-reported dysphagia severity scales

**DHI**	**Normal (n = 6)**	**Mild (n = 7)**	**Moderate (n = 39)**	**Severe (n = 5)**	**F(3,53)**	**P**
	**Mean ± SD**	**Mean ± SD**	**Mean ± SD**	**Mean ± SD**		
Physical	6.33 ± 2.34	8.43 ± 4.47	13.95 ± 6.13	23.20 ± 6.57	9.74	> 0.001
Functional	4.00 ± 0.00	5.43 ± 3.78	12.46 ± 6.59	21.20 ± 10.06	9.09	> 0.001
Emotional	5.00 ± 2.10	6.57 ± 3.41	9.90 ± 4.13	18.80 ± 5.22	12.84	> 0.001
Total	15.33 ± 3.27	20.43 ± 6.92	36.31 ± 12.00	63.20 ± 18.14	19.41	> 0.001

SD: Standard deviation; DHI: Dysphagia Handicap Index

To determine the correlation between the subscales and the total P-DHI scale, Pearson’s correlation coefficient was calculated. There was a significant correlation between the total scale and the physical (r = 0.82, P < 0.001), functional (r = 0.87, P < 0.001) and emotional (r = 0.77, P < 0.001) subscales. Furthermore, a significant correlation was observed between the physical and functional subscales (r = 0.53, P < 0.001), physical and emotional subscales (r = 0.46, P < 0.001), and the functional and emotional subscales (r = 0.57, P< 0.001).

## Discussion

In recent years, much attention is given to the application of patient-centered measures for evaluating voice and swallowing disorders.^14^ Focusing on the patient’s self-perception of dysphagia with their medical diagnosis, the therapist can have a broad picture of the patient’s health status which is useful for planning the treatment protocols.^7^ Hence, making such tools to assess various aspects of a disorder is very important and valuable. DHI is a new tool which evaluates physical, functional and emotional aspect of dysphagia and it has excellent validity and reliability. 

The purpose of this study was to evaluate the validity and reliability of the P-DHI in neurological disorders. The results of this study revealed that the P-DHI like other DHI versions has a good content validity.^7^^,^^14^ There were no problems during translation and cross-cultural adaptations as P-DHI had simple and clear items. This indicates the clinical use of the P-DHI as an easy-to-complete tool for assessing dysphagia consequences on the quality of life.

The Cronbach’s alpha coefficient for the total P-DHI and physical, functional and emotional subscales was between 0.71 and 0.88, indicating a good internal consistency of the P-DHI. These findings are in agreement with the original study and with the Arabic version of the DHI.^7^^,^^14^ ICC for original DHI and the Arabic version of DHI were 0.83 and 0.90, respectively.^7^^,^^14^ Similarly, the results of our study indicated that P-DHI has strong test-retest reliability (ICC = 0.98). Based on these findings, it can be stated that the P-DHI has good validity and reliability among patients with neurological disorders. 

The subjects in this study gained the highest mean scores, respectively, on physical, functional, and emotional subscales, in accordance with previous reports.^7^^,^^14^ It is probable that the physical subscale contained items easier to understand by the patients. Another possible explanation would be that they might be more concerned with the physical aspects of their oropharyngeal dysphagia than the other ones.

In this study, we had no comparison between P-DHI and other tools that evaluated quality of life in the patients with dysphagia. Hence, it is recommended that in the future studies concurrent validity should be considered.

## Conclusion

The results of our study indicate that P-DHI is a valid and reliable tool among patients with neurological disorders; as a result, it could be used to evaluate disabling effects of dysphagia on quality of life of these patients.

## Notes:

**How to cite this article:** Barzegar-Bafrooei E, Bakhtiary J, Khatoonabadi AR, Fatehi F, Maroufizadeh S, Fathali M. Validation of the Persian version of the dysphagia handicap index in patients with neurological disorders. Iran J Neurol 2016; 15(3): 128-32**.**

## References

[1] Rofes L, Arreola V, Almirall J, Cabre M, Campins L, Garcia-Peris P (2011). Diagnosis and management of oropharyngeal dysphagia and its nutritional and respiratory complications in the elderly. Gastroenterol Res Pract.

[2] Rajaei A, Barzegar Bafrooei E, Mojiri F, Nilforoush MH (2014). The Occurrence of laryngeal penetration and aspiration in patients with glottal closure insufficiency. ISRN Otolaryngol.

[3] Ekberg O, Hamdy S, Woisard V, Wuttge-Hannig A, Ortega P (2002). Social and psychological burden of dysphagia: its impact on diagnosis and treatment. Dysphagia.

[4] Carlsson S, Ryden A, Rudberg I, Bove M, Bergquist H, Finizia C (2012). Validation of the Swedish M. D. Anderson Dysphagia Inventory (MDADI) in patients with head and neck cancer and neurologic swallowing disturbances. Dysphagia.

[5] Garcia-Peris P, Paron L, Velasco C, de la Cuerda C, Camblor M, Breton I (2007). Long-term prevalence of oropharyngeal dysphagia in head and neck cancer patients: Impact on quality of life. Clin Nutr.

[6] Nguyen NP, Frank C, Moltz CC, Vos P, Smith HJ, Karlsson U (2005). Impact of dysphagia on quality of life after treatment of head-and-neck cancer. Int J Radiat Oncol Biol Phys.

[7] Silbergleit AK, Schultz L, Jacobson BH, Beardsley T, Johnson AF (2012). The Dysphagia handicap index: development and validation. Dysphagia.

[8] Verdonschot RJ, Baijens LW, Serroyen JL, Leue C, Kremer B (2013). Symptoms of anxiety and depression assessed with the hospital anxiety and depression scale in patients with oropharyngeal dysphagia. J Psychosom Res.

[9] Zhang L, Huang Z, Wu H, Chen W, Huang Z (2014). Effect of swallowing training on dysphagia and depression in postoperative tongue cancer patients. Eur J Oncol Nurs.

[10] Chen AY, Frankowski R, Bishop-Leone J, Hebert T, Leyk S, Lewin J (2001). The development and validation of a dysphagia-specific quality-of-life questionnaire for patients with head and neck cancer: the M. D. Anderson dysphagia inventory. Arch Otolaryngol Head Neck Surg.

[11] Woisard V, Andrieux MP, Puech M (2006). Validation of a self-assessment questionnaire for swallowing disorders (Deglutition Handicap Index). Rev Laryngol Otol Rhinol (Bord).

[12] Gustafsson B, Tibbling L (1991). Dysphagia, an unrecognized handicap. Dysphagia.

[13] Speyer R, Heijnen BJ, Baijens LW, Vrijenhoef FH, Otters EF, Roodenburg N (2011). Quality of life in oncological patients with oropharyngeal dysphagia: Validity and reliability of the Dutch version of the MD Anderson Dysphagia Inventory and the Deglutition Handicap Index. Dysphagia.

[14] Farahat M, Malki KH, Mesallam TA, Bukhari M, Alharethy S (2014). Development of the Arabic Version of Dysphagia Handicap Index (DHI). Dysphagia.

[15] Beaton DE, Bombardier C, Guillemin F, Ferraz MB (2000). Guidelines for the process of cross-cultural adaptation of self-report measures. Spine (Phila Pa 1976).

[16] Logemann JA, Veis S, Colangelo L (1999). A screening procedure for oropharyngeal dysphagia. Dysphagia.

[17] Ansari NN, Naghdi S, Hasson S, Valizadeh L, Jalaie S (2010). Validation of a Mini-Mental State Examination (MMSE) for the Persian population: A pilot study. Appl Neuropsychol.

